# Permissive hypercapnia for severe acute respiratory distress syndrome in immunocompromised children: *A single center experience*

**DOI:** 10.1371/journal.pone.0179974

**Published:** 2017-06-20

**Authors:** Hans Fuchs, Nicola Rossmann, Manuel B. Schmid, Manfred Hoenig, Ulrich Thome, Benjamin Mayer, Daniel Klotz, Helmut D. Hummler

**Affiliations:** 1 Center for Pediatrics, Department of Neonatology and Pediatric Intensive Care, Medical Center – Albert Ludwig University of Freiburg, Faculty of Medicine, Freiburg, Germany; 2 Division of Neonatology and Pediatric Critical Care, Department for Pediatrics and Adolescent Medicine, Ulm University, Ulm, Germany; 3 Department of Neonatology, University Hospital Zurich, University of Zurich, Zurich, Switzerland; 4 Oncology and stem cell transplantation, Department for Pediatrics and Adolescent Medicine, Ulm University, Ulm, Germany; 5 Division of Neonatology, University Hospital of Leipzig, Leipzig, Germany; 6 Institute of Epidemiology and Medical Biometry, Ulm University, Ulm, Germany; National Yang-Ming University, TAIWAN

## Abstract

**Background:**

Controlled hypoventilation while accepting hypercapnia has been advocated to reduce ventilator-induced lung injury. The aim of the study was to analyze outcomes of a cohort of immunocompromised children with acute respiratory distress syndrome (ARDS) ventilated with a strategy of stepwise increasing PCO_2_ targets up to 140 mm Hg.

**Methods:**

Retrospective analysis of outcomes of a cohort of children with oncologic disease or after stem cell transplantation and severe respiratory failure in comparison with a historical control cohort.

**Results:**

Out of 150 episodes of admission to the PICU 88 children underwent invasive mechanical ventilation for >24h (overall survival 75%). In a subgroup of 38 children with high ventilator requirements the PCO_2_ target ranges were increased stepwise. Fifteen children survived and were discharged from the PICU. Severe pulmonary hypertension was seen in two patients and no case of cerebral edema was observed. Long term outcome was available in 15 patients and 10 of these patients survived without adverse neurological sequelae. With introduction of this strategy survival of immunocompromised children undergoing mechanical ventilation for >24h increased to 48% compared to 32% prior to introduction (historical cohort).

**Conclusions:**

A ventilation strategy incorporating very high carbon dioxide levels to allow for low tidal volumes and limited inspiratory pressures is feasible in children. Even severe hypercapnia may be well tolerated. No severe side effects associated with hypercapnia were observed. This strategy could potentially increase survival in immunocompromised children with severe ARDS.

## Introduction

Permissive hypercapnia is a ventilation strategy to allow for an unphysiologically high partial pressure of carbon dioxide (PCO_2_) to permit lung protective ventilation with low tidal volumes. Current guidelines recommend the concept of low tidal volume ventilation and permissive hypercapnia for patients with sepsis, acute respiratory distress syndrome (ARDS) or acute on chronic respiratory failure [1;2]. However, there is insufficient clinical data which levels of PCO_2_ can safely be allowed for, and there are no data available if such a strategy translates into a survival benefit.

In several models hypercapnic acidosis was associated with benefits on lung and distant organs apart from the reduction of ventilation parameters: In *in vivo* and *ex vivo* models for ventilator induced lung injury [3–5], ARDS [6;7], ischemia reperfusion injury [8;9] and sepsis [10] therapeutic hypercapnia through inspired carbon dioxide attenuated lung injury, as measured by gas exchange, reduced cytokine release, lung edema formation and histological lung injury. Differential effects of hypercapnia on bacterial infection i.e. pneumonia have been observed [11]: While hypercapnia can prevent lung damage in established and treated pneumonia, increased bacterial loads and deteriorating lung function was observed in evolving untreated pneumonia [12;13]. The clinical impact of potential negative effects of hypercapnic acidosis like impaired wound healing [14;15] or decreased fluid resorption from the lung [16] is unclear. Nevertheless, all these effects may add on to a possible benefit of a low pressure, low tidal volume strategy in regard to lung protection.

Very few clinical trials have addressed permissive hypercapnia until present: In the late 90s Amato et al. compared in a randomized controlled trial a lung protective strategy including low tidal volumes and permissive hypercapnia with standard therapy [17]. They observed a reduction in mortality; however effects of low tidal volumes and hypercapnia were not differentiated. In 2000 the ARDS Network trial demonstrated superiority of a low tidal volume strategy on mortality [18]. A secondary analysis of this trial showed lung protective properties of mild hypercapnic acidosis in the subgroup of injuriously ventilated patients [19]. Very recently, in contrast, the concept of permissive hypercapnia was challenged by results from a large prospective observational database. In adults with ARDS an increased mortality was associated with the presence of hypercapnia, even after correction for the more severe ARDS in these patients [20].

Stimulated by experimental and clinical data in 2003 we began to use permissive hypercapnia in children with primary immunodeficiency, with oncologic disease and patients after human stem cell transplantation (HSCT) if mechanical ventilation otherwise would have been regarded as too injurious and because we considered extracorporal membrane oxygenation (ECMO) in such patients futile.

The aim of this study is to report outcomes, experiences, and the side effects in this unique patient cohort treated with a protocol of stepwise allowing for very high PCO_2_ values, and to compare those results with a historical cohort from our unit.

## Materials and methods

This retrospective cohort study was approved by the ethics committee of the University of Ulm, Germany (No. 230/11).

All admissions of immunocompromised patients to the level three pediatric intensive care unit, Department for Pediatrics and Adolescent Medicine, Ulm University between 1996 and 2010 were identified by review of admission logs of the Pediatric Intensive Care Unit (PICU). Immunocompromised patients were defined as primary immunodeficiency, ongoing chemotherapy for malignancy or ongoing treatment with allogenic HSCT. Medical records of included patients were reviewed.

First, patients invasively ventilated with target arterial PCO_2_>60 mm Hg for >24h were identified from the entire cohort. In case of repeated readmissions to the PICU including invasive ventilation only the last (sometimes fatal) episode was included.

In November 2003 the concept of permissive hypercapnia was introduced in an individual patient based on experimental data as an individual treatment attempt with success and, on from that time point, it was applied to all following patients with respiratory failure and high ventilator settings to maintain a tidal volume of <6 ml/kg and a peak inspiratory pressure (PIP) of <30 mbar, if possible. In general hypercapnia was permitted early after intubation with a lower range (i.e. 50–70 mm Hg) and subsequently increased by 10–20 mm Hg/per day up to ranges of max. 120–140 mm Hg if necessary. Other unit policies to protect the lung included noninvasive ventilation for less severe respiratory distress, higher ventilation rates during invasive ventilation adjusted to individual lung mechanical properties, avoidance of air trapping and high frequency oscillatory ventilation as rescue strategy. These strategies had been introduced many years before introduction of the concept of permissive hypercapnia. Patient charts were analyzed in detail: All available information on patient history, clinical course, ventilation parameters, laboratory values, X-ray images, echocardiography as well as outcome parameters were collected. The PRISM24 III score was determined at the day of intubation according to [21]. The PELOD score was determined throughout stay in the PICU according to [22]. Long term outcome was taken from the medical records of the last follow up visit.

For comparison of outcome variables, a historical cohort from the 7 years before (1/1996-10/2003) was compared to the Permissive Hypercapnia cohort (11/2003-12/2010). All episodes of admissions of immunocompromised children were identified between 1/1996 and 12/2010. Patient data of interest were identified from medical records and are given below. From both cohorts a subcohort was defined, which included immunocompromised patients with invasive ventilation for >24h. Again, if patients were repeatedly admitted and underwent mechanical ventilation more than once, only the last episode of admission (expected to be associated with the highest risk for death) was included.

Statistics: Data were processed in Excel (Microsoft, Redmond, WA). Data were analyzed using SigmaStat V2.03 (Systat Software, San Jose, California) or SAS V9.2 (SAS Institute,Cary, N.C.). Data are reported as median and interquartile ranges or numbers and percent. Statistical analysis of numerical or categorical data was performed using a t-test, u-test, Χ2-test or fishers exact test as appropriate. Comparison of groups with repeated measurements were analyzed by using a mixed model (SAS). Survival analysis was done using the the LogRank test.

## Results

Thirty eight immunocompromised patients undergoing mechanical ventilation with moderate to high PCO_2_ target ranges (>60 mm Hg) were identified. Patient data are given in Table 1. ARDS was moderate in 3 patients and severe in all others [23]. Survivors had a lower PRISM24 III at time of intubation and lower PELOD scores during the stay. There was no difference in ventilation settings and parameters of gas exchange at the time of intubation between surviving and non-surviving patients.

**Table 1:** Patient data (Permissive hypercapnia cohort).

	All n = 38	Survivors n = 15	Non-survivors n = 23	p
**Age (years)**	3.83(0.57;9.2)	3.52 (0.59;8.44)	3.89 (0.59;8.49)	0.88
**Weight (kg)**	12.6 (7.1;20.9)	12.6(7.4;25.9)	12.7(6.54;20.7)	0.77
**Gender (f/m)**	12/26	4/11	8/15	0.47
**Underlying disease**				
Immunodeficiency	n = 16(42)	n = 7(47)	n = 9(39)	0.9
Leukemia	n = 10(26)	n = 4(27)	n = 6(26)	1.0
Solid tumor	n = 2(5)	n = 1(7)	n = 1(4)	1.0
Other	n = 10(26)	n = 3(20)	n = 7(30)	0.7
**Stem cell transplantation**	23(61)	11(73)	12(52)	0.33
HLA identical	10	6	4	
HLA haploidentical	13	5	8	
**Scores**				
PRISM 24*	16(10;24)	12(5;16)	20(12;27)	0.001
Pelod-Score	43(32;52)	32(23;42)	44(34;53)	0.004
**Intubation**				
paO_2_/FiO_2_ (mm Hg)	84(64;110)	81(64;124)	84(62;110)	0.73
PIP (cm H_2_O)	29(24;33)	32(24;36)	28(24;33)	0.68
PEEP(cm H_2_O)	10(8;12)	10(8;12)	9(7;12)	0.23
FiO_2_	0.78(0.6;1.0)	0,7(0.6;0.85)	1.0(0.6;1.0)	0.17

*day of intubation, median (1.;3. quartile)

**Fig 1:**
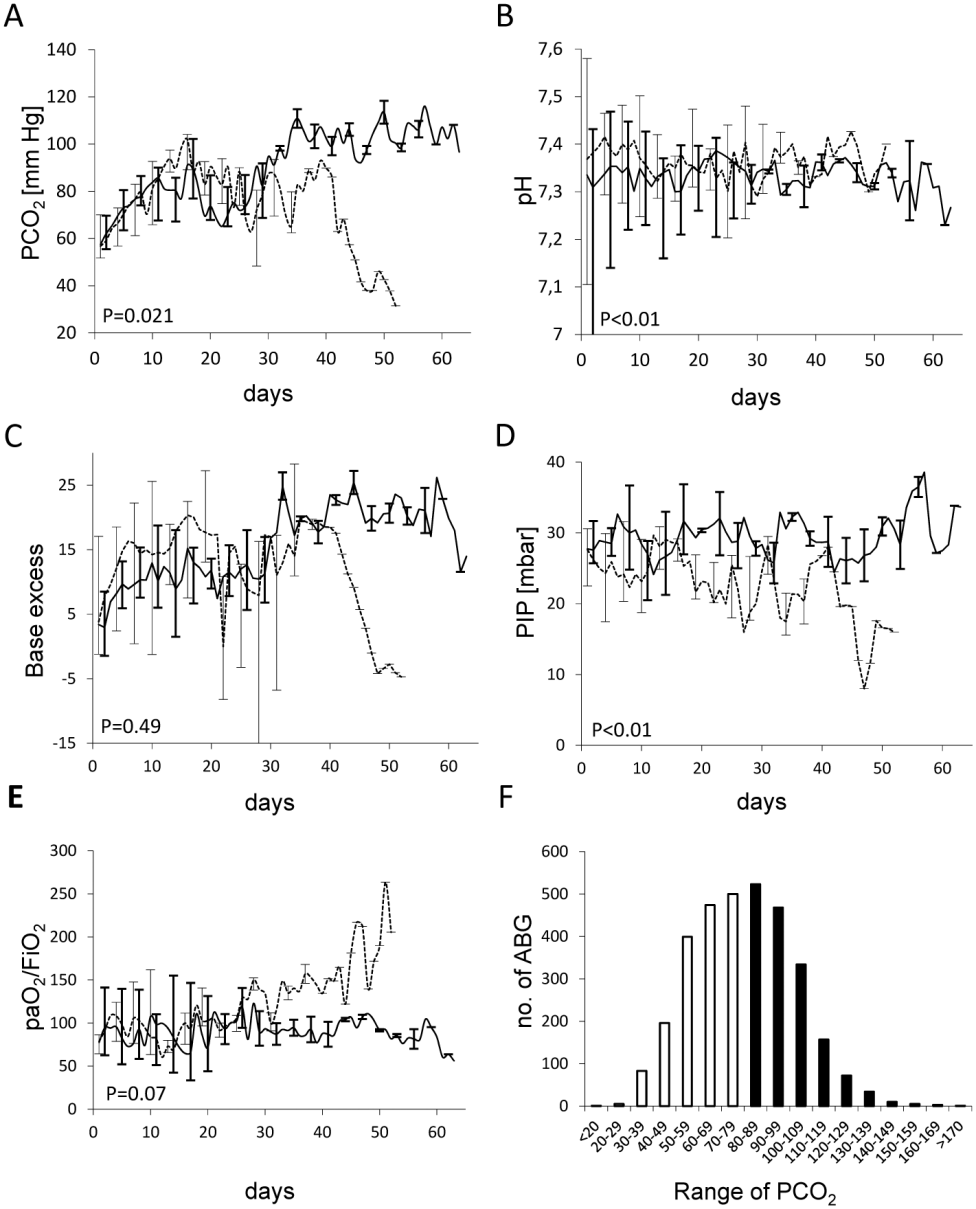
Blood gas result, ventilation parameters, and number of blood gases of the cohort of immunocompromised children (n = 38) ventilated with allowance of severe hypercapnia. Dashed line = survivors; solid line = non-survivors. Median and interquartile ranges are given.

Course of PCO_2_ is given in Fig 1A for surviving and non-surviving patients of the cohort ventilated at high PCO_2_ targets. The highest individual PCO_2_ target range aimed for by the clinical team was 120 to 140 mm Hg. Cumulative PCO_2_ values measured in the entire cohort over time is given in Fig 1F. By unit policy the target range for PCO_2_ was gradually increased by 10–20 mm Hg per day (Fig 1A). We observed a rapid metabolic compensation for respiratory acidosis, therefore, pH remained within acceptable limits despite very high PCO_2_ levels in the majority of patients (Fig 1B and 1C). However, there was a high variability in PCO_2_ levels (Fig 1B and 1C). PIP was targeted to remain below 30 cm H_2_O (Fig 1D). Initial ventilator rate was 35 (30;47)/min (median (interquartile range)) 2-4h after intubation.

**Fig 2:**
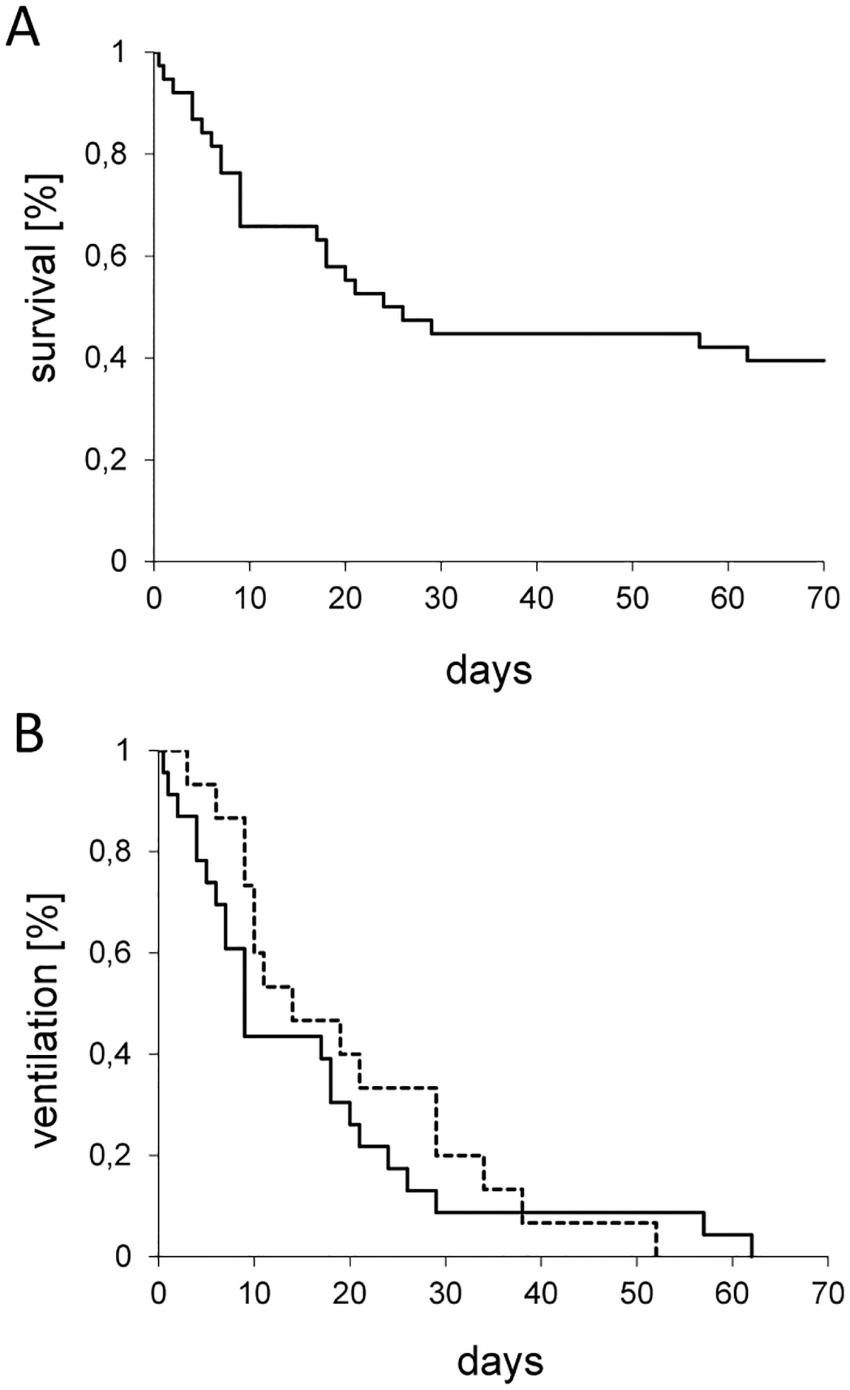
Survival and length of ventilation of a cohort of immunocompromised 38 children exposed to severe permissive hypercapnia. (A) Survival plot and (B) length of ventilation. Dashed line = survivors; solid line = non-survivors.

Overall 15 out of 38 patients survived (39%; Fig 2A). Length of invasive ventilation was 13 (9; 29) days for the survivors and 9 (6;21) days for the non-surviving patients (Fig 2B). Notably, one patient was successfully extubated after 52 days of invasive ventilation.

We observed pneumothoraces in 6/38 patients and pneumomediastinum in 4/38 patients. Pulmonary hypertension was observed in 7/38 patients and was graded severe in two patients. Occurrence of pulmonary hypertension was always associated with death. Abnormal cardiac contractility was seen in nine patients. It was severe in two patients. Patients that developed renal failure had a significantly lower survival than patients without renal failure (Table 2).

**Table 2:** Adverse events (Permissive hypercapnia cohort).

	All n = 38	Survivors n = 15	Non-survivors n = 23	p
**Pneumothorax**	n = 6	n = 1	n = 5	0.37
**Mediastinal emphysema**	n = 4	n = 2	n = 1	0.55
**Pulm. hypertension**	n = 7	-	n = 7	0.029
mild	n = 3	-	n = 3	
medium	n = 2	-	n = 2	
severe	n = 2	-	n = 2	
**Abnormal cardiac function**	n = 9	n = 2	n = 7	0.27
mild	n = 4	n = 1	n = 3	
medium	n = 3	n = 1	n = 2	
severe	n = 2	-	n = 2	
**Nosocomial sepsis**	n = 10	n = 3	n = 7	0.71
**Ventilator-associated pneumonia**	n = 2	-	n = 2	0.51
**Renal failure**	n = 19	n = 4	n = 15	0.046
**Other adverse events**		n = 1 seizure	n = 1 seizure	1.0
			n = 1 pulm. hemorrhage	

Long term outcome of surviving patients is given in Table 3. There were further 6 late deaths not associated with the episode of respiratory failure. Neurological outcome of the survivors after treatment with severe permissive hypercapnia was normal in 10 survivors. Mild learning difficulties were found in two patients and three patients had moderate or severe mental impairment (Table 3). Neurologic impairment was present in two of them prior to admission or might be related to an large underlying brain tumor in one patient.

**Table 3:** Long term outcome (Permissive hypercapnia cohort).

Pat.	Neurological outcome	Pulmonary outcome	other	alive	Cause of death (time to death in days)
1	Mild learning difficulties	Restrictive pulmonary disease		y	n.a.
4	Mild learning difficulties	Lung fibrosis 24h- O_2_ dependence	CMV-reactivation/immunosuppression	n	Lung fibrosis (95)
5	Normal	None	Erythrodermia, paresthesias Abn. liver function tests thrombocytopenia	n	Sepsis/pneumonia (412)
6	Moderate learning difficulties	Normal	Hypogonadism, herpes zoster	y	n.a.
8	Normal	Normal		n	Sepsis, DNR (6)
10	Normal	Normal	Craniosynostosis	y	n.a.
12	Normal	Normal		n	Sepsis (210)
19	Normal	Normal	Mitral valve insufficieny	y	n.a.
21	Moderate learning difficulties	Chronic lung disease, 24h O_2_ dependence	Chron GVHD of liver and skin, seizures, art Hypertension, EBV-Lymphoma	n	EBV (1229)
22	Normal	Mild obstructive pulmonary disease	Hemiparesis, Dermal mycosis, intracranial calcification, short stature	y	n.a.
24	Normal	Moderate obstructive pulmonary disease	Dermatitis, seizures	y	n.a.
27	Normal			y	n.a.
30	Normal		Microcephaly, normal IQ, specific language impairment	y	n.a.
34	Mental impairment	Recurrent pneumonias	Gastroesophageal reflux	n	Progress of brain tumor (43)
38	Normal	Normal		y	n.a.

**Fig 3:**
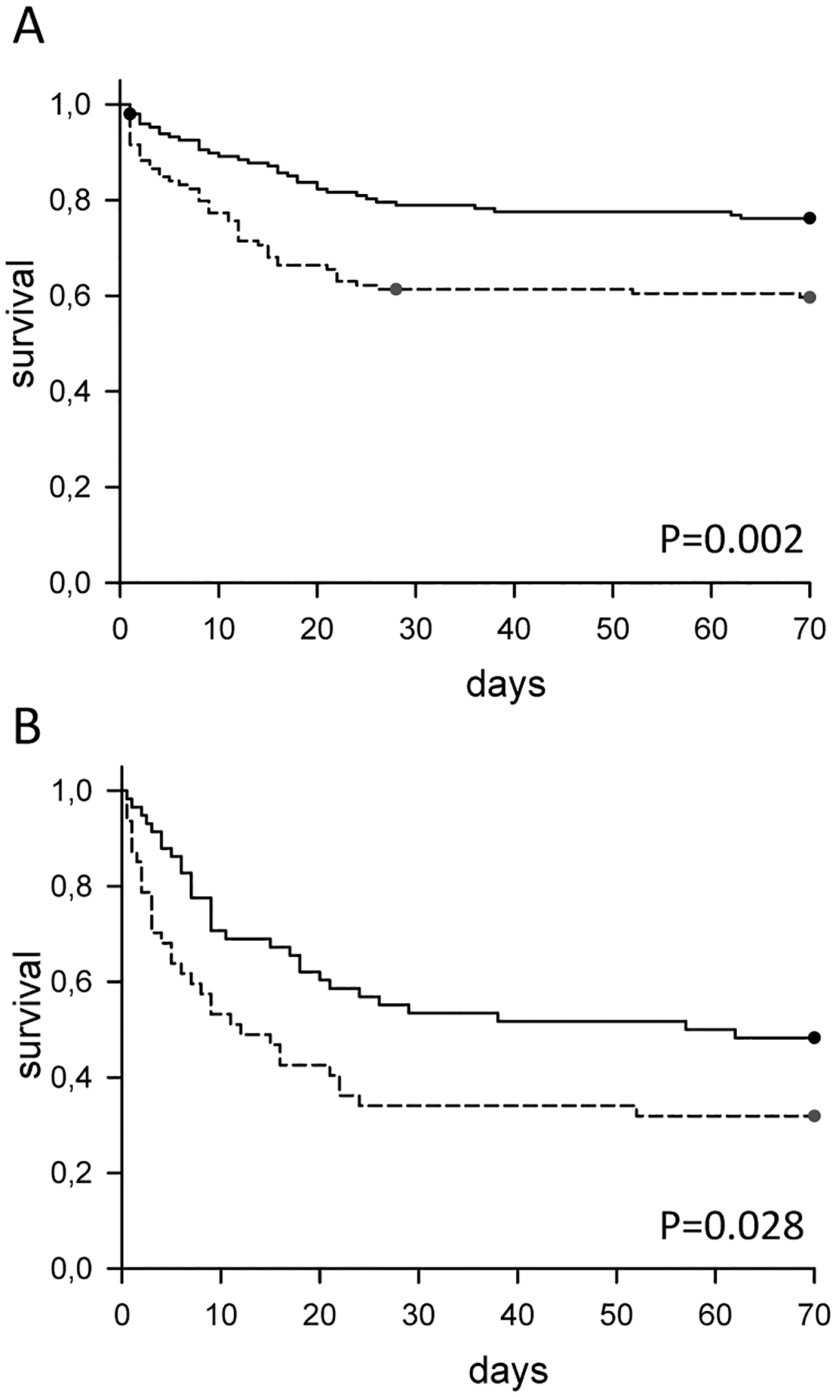
Comparison of the permissive hypercapnia period (Cohort A) with a historical period (Cohort B). (A) Survival plots of all episodes of admission of immunocompromised children to the PICU. (B) Survival plots of immunocompromised children ventilated >24h. Solid line = Permissive hypercapnia cohort; dashed line = historical cohort.

To estimate the impact of this strategy of allowing for extreme hypercapnia on patient survival we compared this cohort to a seven year historical cohort prior to introduction of the permissive hypercapnia concept. Data of the two cohorts are given in Tables 4 and 5. Despite the tendency towards more severe respiratory failure (as assessed by the PaO_2_/FiO_2_ gradient), chance of survival was significantly higher in the permissive hypercapnia cohort looking at all episodes of admissions of immunocompromised patients to the PICU (Fig 3A) as well as looking at children ventilated ≥24h (Fig 3B).

**Table 4:** Comparison of the recent permissive hypercapnia period with a historical period. All admissions of immunocompromised patients to the PICU.

	Cohort A 3.2003–12.2010	Cohort B 1.1996–2.2003	p
**Admissions (n)**	150	121	
**Patients (n)**	101	101	
**Any invasive ventilation;n (%)**	88 (59)	60 (50)	0.26
**Age (y)**	5.1(1.3;12.5)	4.0(1;12.5)	0.41
**Gender (male)**	100(71)	76(60)	0.59
**Length of PICU stay (d)**	3(1;12)	5(1;12)	0.23
**Survival (%)**	113 (75)	71(59)	0.005

**Table 5:** Comparison of the recent permissive hypercapnia period with a historical period. Patients ventilated ≥24h*.

	Cohort A 3.2003–12.2010	Cohort B 1.1996–2.2003	p
**Patients (n)**	58	47	
**Age (y)**	3.8(0.7;8.8)	2.7(0.5;12.2)	0.88
**Gender (male)**	41(71)	28(60)	0.26
**Type of disease**			
Immunodeficiency	24(41)	20(43)	0.93
Leukemia	13(22)	12(26)	0.89
Solid tumor	7(12)	2(4)	0.28
Other	14 (24)	13(28)	0.85
**HSCT (%)**	32 (55)	33(70)	0.17
**PRISM III score**	17(9;21)	16(11;20)	0.93
**paO**_**2**_**/FiO**_**2**_ **(mm Hg)**	104(67;160)	122(100;166)	0.057
**pH**	7.35(7.26;7.43)	7.4(7.3;7.44)	0.25
**PIP (mbar)**	30(24;34)	26(24;33)	0.52
**PEEP (mbar)**	9(6;11)	8(5;10)	0.02
**Ventilator rate (/min)**	35(27;44)	35(25;45)	0.94
**Permissive hypercapnia (n)**	38(66)	-(-)	
**Length of PICU-stay (d)**	13.5(4.3;24)	9(3.5;16)	0.12
**Survival (%)**	28 (48)	15(32)	0.135

*In case of repeated episodes only the last episode per patient is included *HSCT* human stem cell transplantation, *PIP* positive inspiratory pressure, *PEEP* positive end-expiratory pressure

## Discussion

Herein, we report the single center treatment results of immunocompromised children with severe ARDS treated according to a protocol of stepwise increase of PCO_2_ to allow for lung protective ventilation with low tidal volumes and limited inspiratory pressures. Our findings indicate that: first the feasibility of such a strategy, second the limited rate of severe side-effects like pulmonary hypertension or neurological complications, third we provide some long term outcome data and, furthermore, there is some evidence that such a strategy might result in higher survival rates.

Respiratory failure during severe immunosuppression i.e. oncologic disease and after HSCT remains a major threat. More recent reports indicate improved PICU survival rates of 59% (6 month survival of 43%) [24]; 58% [25]; and 58% [26] of admissions of children with HSCT or oncologic disease requiring any mechanical ventilation. At a first glance, these results seem to match well with our results, however, given the high proportion of HSCT patients in our cohort, the exclusion of patients ventilated <24h and including only the last episode of ventilation if patients were readmitted repeatedly, our results appear even more favorable. For example, survival through their last PICU discharge decreased to 39% in the study from Cincinnati for HSCT patients who needed any mechanical ventilation [26]. Another recent study from Seattle differentiated HSCT patients ventilated for >24h and survival was as low as 25% in this subcohort [27]. Therefore, a survival rate of almost 40% appears to be rather high, which might support our use of hypercapnia.

Various direct effects of hypercapnic acidosis have been described: these include attenuation of inflammatory mediators i.e. attenuation of neutrophil function, antioxidative properties, reduction in proinflammatory cytokine levels and apoptosis. These effects have translated into benefits in various preclinical models like ischemia-reperfusion or ventilation induced lung injury [3;4;8;9]. Differential or negative effects were noted in sepsis/pneumonia and organ recovery models [13;15]. As buffering reverses some of the effects [28] it is not clear if these direct effects of acidosis become meaningful in our patients: We observed rapid metabolic compensation in our cohort of patients eliminating severe acidosis quickly. Nevertheless, our goal was to allow for a strategy of applying tidal volumes as low as possible at rather high ventilator rates depending on the lung mechanical properties. This goal was achieved by increasing the PCO_2_ target ranges daily to maximum levels. Recent experimental [7] and clinical data [29;30] further support the use of ultraprotective low tidal volumes in the setting of ARDS. However, in these publications progressive hypercapnia was avoided by extracorporal CO_2_ elimination. This was disregarded in our patient subset because extracorporeal membrane oxygenation is considered futile in hematologic disease [31].

Unwanted side effects have to be taken into account when using a strategy of allowing for extreme hypercapnia. Early ARDS may be associated with acute cor pulmonale in adults in about 20% of patients [32]. This may add to the pulmonary vasoconstriction caused by hypercapnia [33] and a PCO_2_>60 mm Hg was shown to be an independent risk factor for acute cor pulmonale [32]. We observed pulmonary hypertension in a considerable number of patients, however, it was regarded mild or moderate in the majority, as it was found by Cavalho et al. [34]. Interestingly, despite increasing the risk for acute cor pulmonale an increased PCO_2_ was not associated with death in the study of Lherithier et al. [32]. However, the presence of airleaks increased the risk for death 5-fold, suggesting that aiming for maximal lung protection while carefully monitoring right heart cardiac function may finally sum up to the best benefit.

Abnormal cardiac function was seen in roughly a quarter of our patients. Despite some negative effects of hypercapnia on contractility we may expect an increase in cardiac output due to an increase in sympatoadrenal activity [34]. Furthermore, tissue perfusion is directly increased by hypercapnia and oxygen delivery into the tissues is enhanced by facilitated oxygen release owing to a right shift of the oxygen binding curve [35;36]. Our patients frequently had been treated with multiple cardiotoxic antineoplastic drugs prior to their episode of ARDS, therefore, it seems impossible to differentiate the impact of hypercapnia from a preexisting cardiac disease, ARDS or infection with this retrospective study design.

Multiple reports of acute asthma reassure that even excessively high values of hypercapnia can be survived without sequelae [37;38]. However, there are reports where acute exposure to high CO_2_ was associated with cerebral vasodilation, and cerebral edema. Hypercapnia was considered the main causative reason. In our study no patient suffered or died from clinically relevant increased intracranial pressures.

Some children treated with permissive hypercapnia suffered from learning disabilities or even mental impairment long term. Neurologic sequelae after HSCT or severe ARDS in addition to underlying conditions are well recognized. Our data do not allow to determine the exact cause of impairments among these possibilities, however, no episodes of severe hypoxia or resuscitation were documented in these patients. The proportion of impaired survivors, does not seem to be higher in our permissive hypercapnia cohort.

In the very recent prospective observational trial focusing on ARDS in adults the concept of permissive hypercapnia was challenged, as presence of hypercapnia was associated with increased mortality in this trial [20]. These findings remained significant even after correction for the more severe lung disease in the subgroup of hypercapnia patients as evidenced by a lower paO2/FiO2. In the hypercapnia cohort relevant higher ventilation settings (PIP/PEEP) had been used compared to patients without hypercapnia and cardiovascular failure was observed in 74% of these patients. This is in contrast to the observation in our cohort of children where the risk for cardiovascular failure was much lower.

To unravel potential benefits from our ventilation strategy we compared the mortality within 7 years after introduction of the permissive hypercapnia concept with the 7 preceding years. However, patient data including a severity of illness score and parameters of gas exchange showed a trend towards more severe lung disease in the latter cohort. Nevertheless, survival in patients after introduction of the strategy of permissive hypercapnia ventilated >24h increased by 16%. Obviously, we cannot be sure if this increased survival rate is due to the ventilation policy only. Advances in survival rates in HSCT intensive care patients over the years have also been observed by other centers [25;26]. However, a recent meta-regression analysis challenges this impression for the subset of ventilated patients [39]. Furthermore, permissive hypercapnia is frequently incorporated in the new concepts of intensive care therapy, therefore, may have contributed to some of the improvements seen by others [25;26]. Anyway, there is considerable dispute about the best target ranges of PCO_2_ [40].

The increase in survival is modest. However, mechanical ventilation in ARDS in the setting of immunosuppression is only a supportive measure to buy time for more causative therapies to become effective. Therefore, optimizing ventilation may not alter outcomes if the underlying cause of the ARDS cannot be sufficiently treated. In the survival curves, it seems that the time window for such treatments has opened somewhat in our recent patient cohort even if the final outcome was not altered tremendously. Multiple improvements in all aspects of care will be necessary to achieve better results in respiratory failure after bone marrow transplantation or antineoplastic chemotherapy.

This study has multiple limitations: First, it is a retrospective study. Comparison with a historical cohort bears numerous risks for bias, although we are not aware of major systematic changes in regard to oncologic or antibiotic therapy between the episodes. Attending physicians of oncology and intensive care did not change during study time. New guidelines for intubation at lower thresholds were introduced at the very end of the second time period [41] and have, therefore, not contributed to the results. Furthermore, hypercapnia was introduced only, if the peak pressures exceeded 30 mm Hg and the daily increase in PCO_2_ targets was roughly 10 mm Hg. However, this was modified depending on the patient and at the discretion of the physician responsible. To minimize selection bias all patients admitted to the PICU during the study period were analysed irrespective of exposure to hypercapnic ventilation. Patient numbers are low in this study, however, even in large transplantation centers the number of such cases is limited. Therefore, large scale randomized trials for this subset of patients will remain challenging. Furthermore, there is a wide variety and complexity of underlying diseases as well as reasons for respiratory failure. By pooling these data, potential beneficial effects might be blurred, as some individual patients might benefit more than others. Therefore, results have to be interpreted cautiously.

Herein we report our experiences with the strategy of consequently increasing PCO_2_ targets in order to limit lung damage from high ventilation pressures by allowing for very high PCO_2_. This concept, so far, has to be regarded as individual treatment attempt in a subset of very sick immunocompromised patients with anticipated high morbidity and high mortality. We think a larger scale multi center randomized trial is urgently warranted to assess effects of such a strategy on outcome and to define side effects.

## Conclusion

A ventilation strategy incorporating very high permissive hypercapnia seems to be feasible in immunocompromised children with severe respiratory failure secondary to ARDS. Even high levels of PCO_2_ are tolerated well. No severe side effects associated with hypercapnia were observed. This strategy potentially could increase survival in immunocompromised children with severe ARDS.

## Abbreviations

*ARDS*: acute respiratory distress syndrome
*HSCT*: human stem cell transplantation
*PCO_2_*: partial pressure of carbon dioxide
*PICU*: pediatric intensive care unit
*PIP*: peak inspiratory pressure
